# Dusky-like is required for epidermal pigmentation and metamorphosis in *Tribolium castaneum*

**DOI:** 10.1038/srep20102

**Published:** 2016-02-01

**Authors:** Chengjun Li, Xiaopei Yun, Bin Li

**Affiliations:** 1 Jiangsu Key Laboratory for Biodiversity and Biotechnology, College of Life Sciences, Nanjing Normal University, Nanjing 210023, China

## Abstract

Dusky-like (Dyl) is associated with the morphogenesis of embryonic denticle, adult sensory bristle and wing hair in *Drosophila melanogaster*. And whether Dyl involved in insect post-embryonic development and its signal transduction are poorly understood. Here, phylogenetic analysis revealed that *dyl* displayed one-to-one orthologous relationship among insects. In *Tribolium castaneum*, *dyl* is abundantly expressed at the late embryonic stage. Tissue-specific expression analysis at the late adult stage illustrated high expression of *dyl* in the fat body and ovary. Knockdown of *dyl* resulted in the defects in larval epidermal pigmentation and completely blocked the transitions from larval to pupal and pupal to adult stages of *T. castaneum*. We further discovered that *dyl* RNAi phenotypes were phenocopied by *blimp-1* or *shavenbaby* (*svb*) silencing, and *dyl* was positively regulated by *blimp-1* through *svb* in *T. castaneum*. These results suggest that Dyl functions downstream of Blimp-1 through Svb for larval epidermal pigmentation and metamorphosis. Moreover, *ftz-f1* was down-regulated after RNA interference (RNAi) suppressing any of those three genes, indicating that Ftz-f1 works downstream of Dyl to mediate the effects of Blimp-1, Svb and Dyl on metamorphosis in *T. castaneum*. This study provides valuable insights into functions and signaling pathway of insect Dyl.

Dusky (Dy), Dusky-like (Dyl) and Miniature were identified as the transmembrane proteins containing a zona pellucid (ZP) domain in *Drosophila melanogaster*^1^. Phylogenetic analysis indicated that they have a common ancestor in *D. melanogaster*^2^. Sequence comparison illustrated that Dy has higher amino acid sequence identity with Dyl (70% amino acid sequence identity) than Miniature (only 45% amino acid sequence identity with Dy) at the ZP domains^3^.

RNA *in situ* hybridization and immunostaining showed that *dy* and *miniature* are expressed in pupal wings by 28 hours after puparium formation (APF), but the expression of *dyl* could not be detected in pupal wings at the same stage^3^. Consistent with their expressions, Dy and Miniature share similar roles for wing morphogenesis in *D. melanogaster*, either of which mutation reduced the size of the whole wings by decreasing the size of individual wing epidermal cell^3^. Different from Dy and Miniature, *dyl* mutant embryos were characterized by very small, unhooked denticles, often with a split extremely^2^. Knocking down *dyl* in adults resulted in stub bristles with pigmentation abnormalities^4^. Recently, it is found that *dyl* mutation caused split, thinned, multiple and often very short hairs and a unique planar cell polarity phenotype of adult wing in *D. melanogaster*^5^. These studies of Dyl only focused on embryonic denticles, adult bristles and wing hairs of *D. melanogaster*, which provides limited information about *dyl* functions in the insect. On the other hand, a confused problem has emerged among the current studies that *dy* is evolutionarily more close to *dyl* than *miniature*, whereas *dy* shows similar expressions and functions with *miniature*. Thus, investigation of the functions of Dyl in the insect will help to clarify the functional relationship among Dyl, Dy and Miniature.

At the transcriptional level, the epidermal expression of *dyl* was abolished in *shavenbaby* (*svb*) mutant embryos of *D. melanogaster*^2^, suggesting that Svb is upstream of Dyl. *Svb* is a key selector gene that integrates Wingless and DER pathways to control epidermis differentiation^6^, and it controls the expressions of *miniature*, *singed* and *forked* in embryonic denticles of *D. melanogaster*^7^. But the functional relationship between *svb* and *dyl* in insects is unclear up to now. Moreover, additional upstream genes and downstream genes of Dyl should be identified in the insect.

Recently, we discovered that not only *dyl* but also *blimp-1* and *ftz-f1* transcripts were down-regulated after RNA interference (RNAi) suppressing the G protein-coupled receptor gene, *methuselah-like1* (*Tcmthl1*) (Accession number: HQ188199, orthologous to *Drosophila mthl5*) of *Tribolium castaneum*^8^, which is involved in lifespan, development, stress resistance and reproduction^9^, suggesting that there might be certain relationship among *dyl*, *blimp-1* and *ftz-f1*. In *D. melanogaster*, *blimp-1* RNAi caused lethality at the pupal stages in most of the observed animals^10^, and elevated expressions of *ftz-f1* from 6 h^11^ to 8 h APF but reduced the expression level of *ftz-f1* by 10 h APF^10^. While, Ftz-f1 was required for cholesterol converted into 20-hydroxyecdysone to control the metamorphosis in *D. melanogaster*^12^ and *Leptinotarsa decemlineata* (Say)^13^. Based on these clues, it is hypothesized that *dyl* might be associated with *blimp-1* and *ftz-f1* and involved in the metamorphosis of insect.

To address these issues, we investigated the function of Dyl in *T. castaneum* by RNAi in this study. Furthermore, we explored how *dyl* relates to Blimp-1, Svb and Ftz-f1. These Results indicate that Dyl, Svb and Blimp-1 are essential for larval epidermal pigmentation and metamorphosis in *T. castaneum*.

## Results

### Dyl is widely distributed and conserved in insects

**Figure 1:**
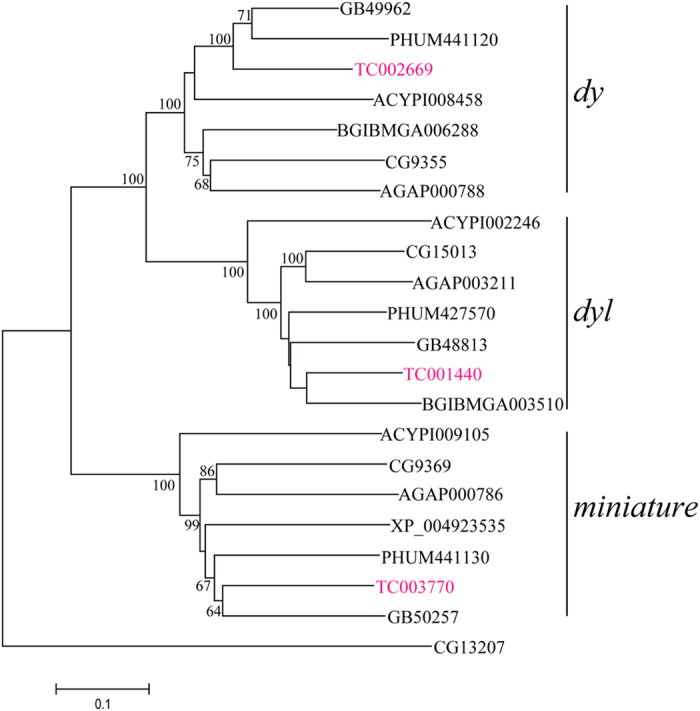
Phylogenetic tree of insect *dy* and *dyl* by Neighbor-joining method. TC NO. indicates the *T. castaneum* gene; GB NO. indicates the *A. mellifera* gene; PHUM NO. indicates the *P. h. humanus* gene; ACYPI NO. indicates the *A. pisum* gene; BGIBMGA NO. and XP_004923535 indicate *B. mori* genes; AGAP NO. indicates *A. gambiae* gene; CG NO. indicates the *D. melanogaster* gene. The tree is rooted by *D. melanogaster* no mechanoreceptor potential A (nompA) (CG13207). The bootstrap value below 60% was removed from phylogenetic tree.

Using *Drosophila dyl* as a reference, 20 homolog genes were obtained from the genome database of *D. melanogaster*, *T. castaneum*, *Anopheles gambiae*, *Bombyx mori*, *Apis mellifera*, *Acyrthosiphon pisum* and *Pediculus humanus humanus*. Phylogenetic analysis revealed that *dyl* has the orthologue in each insect and shows one-to-one orthologous relationship among insects. In addition, its two paralogues, *dy* and *miniature*, also have this orthologous relationship in insects, respectively. And *dyl* is evolutionarily close to *dy* but far away from *miniature* (Figs 1 and S1). Sequence alignment of the proteins encoded by these three genes from *D. melanogaster* and *T. castaneum* also demonstrated that Dyl shares more sequence identity with Dy than Miniature. Structurally, these three genes are coding for the transmembrane proteins containing a ZP domain, respectively (Fig. S2).

### Developmental and tissue expressions of *dyl* in *T. castaneum*

**Figure 2:**
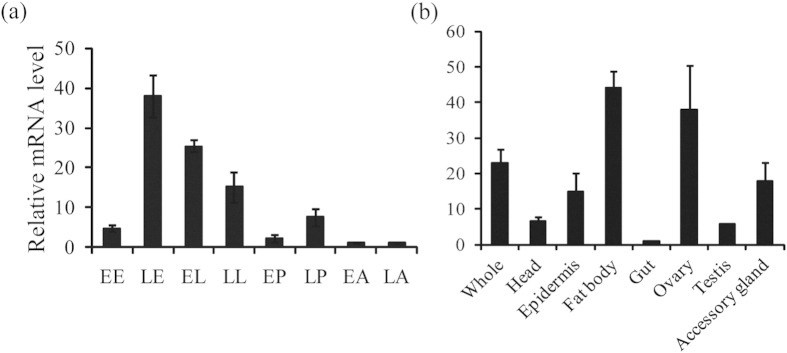
Developmental (**a**) and tissue (**b**) expression patterns of *dyl* in *T. castaneum* by qRT-PCR. EE, early eggs; LE, late eggs; EL, early larvae; LL, last-instar larvae; EP, early pupae; LP, late pupae; EA, early adults; LA, late adults. Tissues were isolated from adults (10-day old). One μg of total RNA from each sample was converted into cDNA for qRT-PCR.

Before functional analysis of *dyl*, we measured mRNA levels of *dyl* at different stages and tissues by quantitative real-time PCR (qRT-PCR) analysis. In *T. castaneum*, *dyl* reached peak expression in the late embryonic stage, while its pupal and adult expression levels were very low (Fig. 2a). Tissue-specific expression analysis at the late adult stage illustrated *dyl* has high expressions in the fat body and ovary, moderate expressions in the epidermis and accessory gland but low expressions in the gut (Fig. 2b).

### *dyl* is essential for larval epidermal pigmentation and metamorphosis of *T. castaneum*

**Figure 3:**
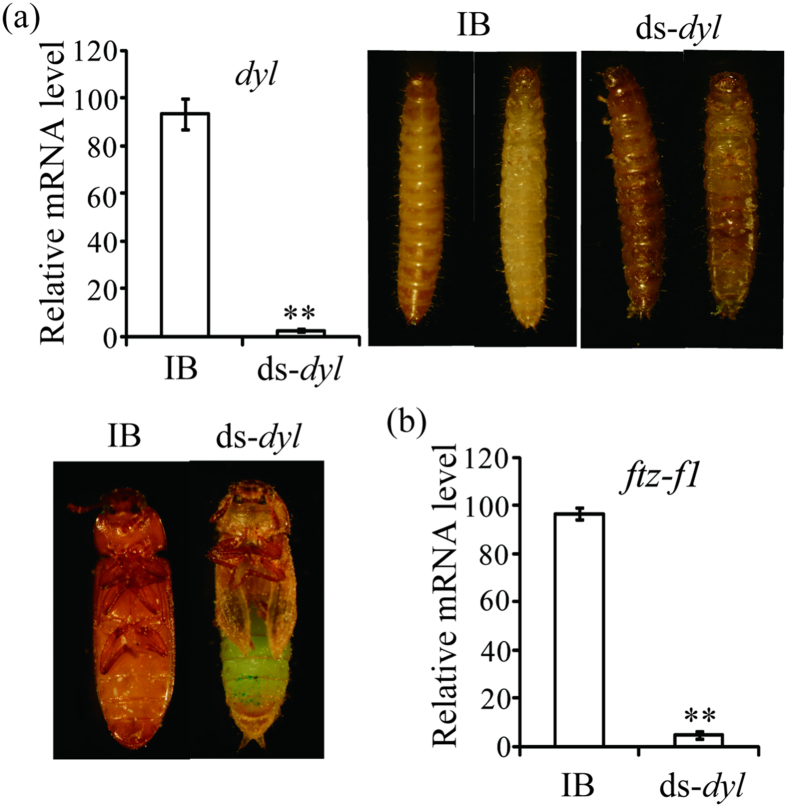
Knockdown of *dyl* affected *T. castaneum* pupation, eclosion (**a**) and reduced *ftz-f1* level (**b**). IB, beetles injected with physiological buffer; ds-*dyl*, beetles injected with *dyl* dsRNA. Three individuals of each group were used to extract total RNA for qRT-PCR analysis.

To clarify the function of *dyl* (Accession number: *TC001440*), dsRNAs of *dyl* were injected into larvae and pupae of *T. castaneum*. The qRT-PCR analysis indicated that the mRNA levels of *dyl* were significantly reduced after dsRNA injection (Fig. 3a). It is observed that all *dyl* dsRNA-treated insects arrested at the larval stage and failed to molt into pupae. The epidermis of these larvae has pigmentation abnormalities. Injection of *dyl* dsRNA into early pupae resulted in developmental arrest prior to eclosion in *T. castaneum* (Fig. 3a). Thus, *dyl* is essential for larval epidermal pigmentation and metamorphosis of *T. castaneum*.

### Dyl positively regulates *ftz-f1* expression in *T. castaneum*

It is shown that reduction of *dyl* strongly inhibited the metamorphosis in *T. castaneum*, and it is likely that there is cross-talk between Dyl and 20-hydroxyecdysone pathways. Therefore, we decided to check the expression of *ftz-f1* that is required for 20-hydroxyecdysone synthesis. As expected, we found that the mRNA level of *ftz-f1* (Accession number: *TC002550*) was down-regulated by nearly 89% compared with IB after *dyl* knockdown (Fig. 3b), suggesting that *dyl* positively regulates *ftz-f1* expression in *T. castaneum*. The result illustrates that Dyl is implicated in 20-hydroxyecdysone pathway.

### Svb plays vital roles in larval epidermal pigmentation, metamorphosis and promotes expressions of *dyl*, *miniature*, *singed* and *forked* in *T. castaneum*

**Figure 4:**
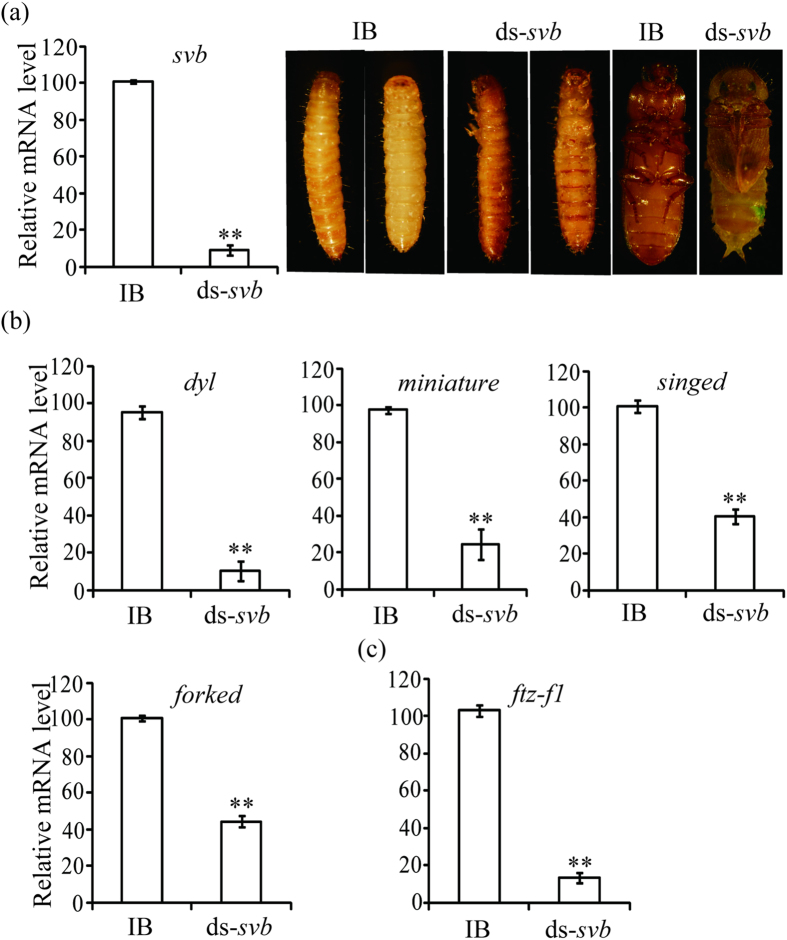
*Svb* RNAi affected pupation and eclosion (**a**) and reduced the expressions of *dyl*, *miniature*, *singed*, *forked* (**b**) and *ftz-f1* (**c**) in *T. castaneum*. IB, beetles injected with physiological buffer; ds-*svb*, beetles injected with *svb* dsRNA. Three larvae or pupae of each group were used to extract total RNA for qRT-PCR analysis.

By RNAi, *svb* (Accession number: *TC008099*) was silenced at larval and pupal stages in *T. castaneum*, respectively. Knock-down of *svb* resulted in arrested development at the larval stage of *T. castaneum*. These larvae have abnormally pigmented epidermis, could not molt into pupae, and then died at larval stage. Pupal RNAi of *svb* also led to the failure to initiate the eclosion of *T. castaneum* (Fig. 4a). In *svb* dsRNA-treated insects, approximately 85% of *dyl* mRNA level was eliminated. It is also observed that more than 50% expressions of *miniature* (Accession number: *TC003770*), *singed* (Accession number: *TC006673*) and *forked* (Accession number: *TC005627*) were declined by *svb* RNAi (Fig. 4b). Thus, *dyl*, *miniature*, *singed* and *forked* are indeed the targets of Svb in *T. castaneum*. Furthermore, *svb* knockdown abolished 89% transcript of *ftz-f1* (Fig. 4c). Taken together, these results indicate that Svb is required for larval epidermal pigmentation and metamorphosis, and promotes the expressions of *dyl*, *miniature*, *singed* and *forked* in *T. castaneum*.

### Blimp-1 is required for metamorphosis, epidermal pigmentation, and positively regulates *svb* and its targets and *ftz-f1* expressions in *T. castaneum*

**Figure 5:**
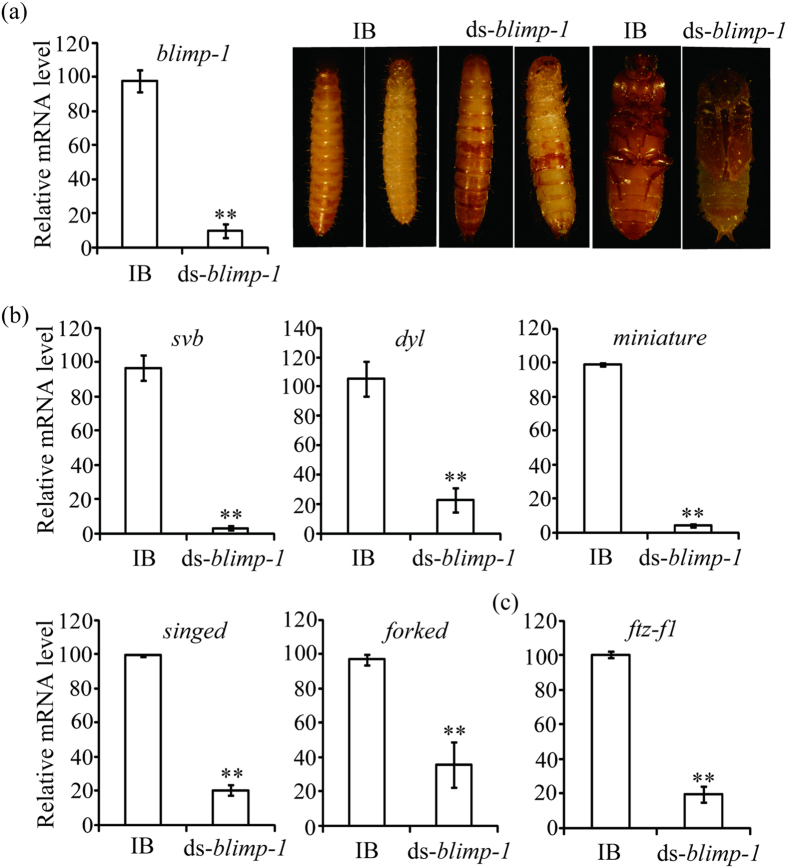
*Blimp-1* RNAi affected pupation and eclosion (**a**) and down-regulated the mRNA levels of *svb* and its targeted genes (**b**) and *ftz-f1* (**c**) in *T. castaneum*. IB, beetles injected with physiological buffer; ds-*blimp-1*, beetles injected with *blimp-1* dsRNA. Three larvae or pupae of each group were used to extract total RNA for qRT-PCR analysis.

Injections of dsRNAs for *blimp-1* (Accession number: *TC014741*) into larvae or pupae depleted about 90% mRNA of itself in *T. castaneum*. Reduced *blimp-1* levels caused developmental arrest at the larval stage of *T. castaneum*. The treated larvae with abnormally pigmented epidermis could not molt into pupae. Injection of *blimp-1* dsRNAs into pupae led to the failure to start the eclosion (Fig. 5a). By qRT-PCR, we demonstrated that *blimp-1* knockdown significantly reduced mRNA levels of *svb* and its targets, and *ftz-f1* in *T. castaneum* (Fig. 5b,c). Overall, Blimp-1 is also essential for larval epidermal pigmentation, metamorphosis, and positively modulates expressions of *svb* and its targets in *T. castaneum*.

## Discussion

In this study, we reveal that *dyl*, *svb* and *blimp-1* are required for larval epidermal pigmentation and metamorphosis of *T. castaneum* (Figs 3a, 4a and 5a). At the transcriptional level, *dyl* positively regulated *ftz-f1* expression but was modulated by *blimp-1* and *svb* (Figs 3, 4, 5).

In *D. melanogaster*, *dyl* was detected in trichome cells of embryo epidermis but not pupal wings^2^,^3^. While, *dyl* mutation or knockdown resulted in very small and unhooked denticles of embryo^2^, stub bristles with pigmentation abnormalities^4^, split, thinned, multiple and often very short hairs and a unique planar cell polarity phenotype of adult wing^5^ in *D. melanogaster*. In this study, *dyl* also showed high expressions in the late embryonic stage and low expressions in the early pupal stage of *T. castaneum* (Fig. 2a). However, RNAi knocking down *dyl* at the late larval and early pupal stages caused the defects in larval epidermal pigmentation and metamorphosis of *T. castaneum* (Fig. 3). Moreover, parental RNAi of *dyl* led to significant embryonic lethal effect in *T. castaneum*, indicating that *dyl* also played some key roles in embryo development of *T. castaneum*, which may be much more critical than that of it in *D. melanogaster*. Tissue-specific analysis revealed that *dyl* is highly expressed in the fat body and ovary of adult in *T. castaneum* (Fig. 2b), supporting the embryonic lethal effect induced by parental RNAi of *dyl*. Thus, it seems that *dyl* shares some common functions between *D. melanogaster* and *T. castaneum*, but has occurred functional shift between them.

In *D. melanogaster*, the epidermal expression of *dyl* was abolished or reduced in *svb* mutant embryos^2^, indicating that *dyl* is positively regulated by *svb*. In addition to *dyl*, the expressions of *miniature*, *singed* and *forked* were also controlled by *svb* in *D. melanogaster*^7^. In *T. castaneum*, *svb* knockdown reduced *dyl*, *miniature*, *singed* and *forked* (Fig. 4b), whereas *dyl* silencing showed no effects on *svb* and *forked* expressions (Fig. S3). RNAi of *dyl* or *svb* led to the defects in larval epidermal pigmentation and metamorphosis in *T. castaneum* (Figs 3a and 4a). These results confirm that Svb is upstream of Dyl for larval epidermal pigmentation and metamorphosis.

In *D. melanogaster*, *blimp-1* knockdown showed lethality at pupal stages in most of the observed animals. Many of them eclosed but died shortly thereafter or died during eclosion^10^. Interestingly, silence of *blimp-1* also resulted in the defects of larval epidermal pigmentation and metamorphosis of *T. castaneum* (Fig. 5a), which is similar to *dyl* or *svb* RNAi phenotypes (Figs 3a and 4a). At the transcriptional level, *blimp-1* RNAi reduced the levels of *svb*, *dyl*, *miniature*, *singed* and *forked* in *T. castaneum* (Fig. 5b). Therefore, Dyl is downstream of Blimp-1 through Svb for larval epidermal pigmentation and metamorphosis in *T. castaneum*.

**Figure 6:**
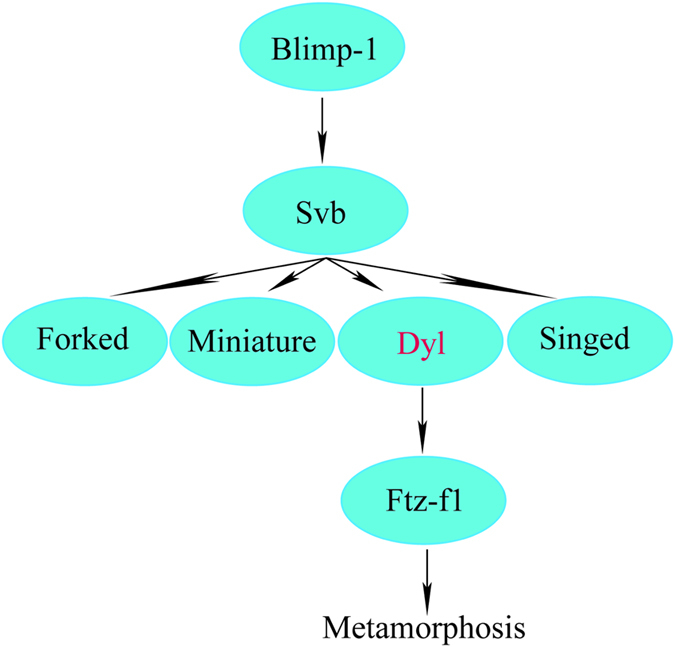
Preliminary regulation model of Dyl in *T. castaneum*.

In *blimp-1* knockdown flies, high-level expressions of *ftz-f1* was detected from 6h^11^ to 8h APF, but the expression level of *ftz-f1* was greatly reduced by 10 h APF, demonstrating that Blimp-1 works as a repressor for premature expression of *ftz-f1* but a activator for latish expression of *ftz-f1*^10^. By qRT-PCR analysis, it is shown that *blimp-1* knockdown down-regulated *ftz-f1* expression at the last-instar larvae of *T. castaneum* (Fig. 5c), suggesting that the expression of *ftz-f1* is positively regulated by *blimp-1* at the last-instar larvae. Likewise, *svb* or *dyl* RNAi also reduced *ftz-f1* level in *T. castaneum* (Figs 3b and 4c). In *T. castaneum*, high expression levels of *dyl* (Fig. 2b) and *ftz-f1*^14^ were detected in the adult fat body and ovary, demonstrating that *dyl* and *ftz-f1* possessed the similar tissue-specific expression pattern. These results indicated that Ftz-f1 works downstream of Dyl, Blimp-1 and Svb in *T. castaneum*. As reported previously, Ftz-f1 was required for cholesterol converted into 20-hydroxyecdysone in *D. melanogaster*^12^ and *L. decemlineata* (Say)^13^. Mutation of *ftz-f1* resulted in arrested development at embryonic, larval and pupal stages in *D. melanogaster*^15^. Reduced levels of *ftz-f1* by RNAi also caused arrested development at larval stage^12^. These studies demonstrate that *ftz-f1* is essential for metamorphosis. Therefore, the effects of Dyl, Svb and Blimp-1 on metamorphosis were modulated by Ftz-f1 in *T. castaneum*. It is concluded that Dyl functions downstream of Blimp-1 through Svb but upstream of Ftz-f1 for metamorphosis (Fig. 6).

Developmental study showed that the presence/absence of *svb* expression ultimately determined the pattern of denticles and dorsal hairs^16^. It was further found that Svb controls the expression of cuticle proteins^17^ and enzymes that increase trichome pigmentation and hardness^7^. These studies reveal the vital roles of Svb for epidermis differentiation. In *T. castaneum*, an obvious defect in epidermal pigmentation was observed in *svb* knockdown larvae (Fig. 4a), confirming the involvement of Svb in epidermal pigmentation. As the target of Svb, *dyl* was only expressed in embryonic tissues that will secrete cuticle, including epidermis, trachea and foregut^2^,^3^. Knocking down *dyl* function disrupted cuticle formation in bristles^4^. As is the case in bristles, hairs lacking *dyl* function also showed abnormality in chitin deposition^5^. In *T. castaneum*, *dyl* silencing caused defects in larval epidermal pigmentation (Fig. 3a). In *D. melanogaster*, Chitinase 6 was identified as a potential candidate of Dyl^5^. It is likely that epidermal pigmentation defects arose from *dyl*, *svb* or *blimp-1* knockdown are associated with chitin deposition. However, how Dyl, Svb and Blimp-1 modulate larval epidermal pigmentation is unclear at present and need to be studied in the future.

Though *dyl*, *dy* and *miniature* of *D. melanogaster* were originated from one ancestral gene^2^, sequence comparison illustrated that Dyl showed more sequence similarities with Dy than Miniature at ZP domains^3^. Based on seven insect genome sequences, our study shows that insect *dyl*, *dy* and *miniature* were divided into three separate clusters of the phylogenetic tree (Figs 1 and S1), providing clues to the functional relationship among these three genes. In *D. melanogaster*, *dy* and *miniature* were detected in pupal wings by 28 hours APF, while *dyl* expression was not detected in pupal wings of the same stage^3^. Functionally, both Dy and Miniature are required for cytoskeletal reorganisation during wing morphogenesis^3^, while *dyl* shows significant effects on embryonic denticles formation^2^, adult bristle cuticle formation^4^ and adult hair integrity and planar cell polarity in *D. melanogaster*^5^. In *T. castaneum*, *dyl* is shown to be required for larval epidermal pigmentation and metamorphosis (Fig. 3a). These results suggest that Dyl is functionally divergent with Dy and Miniature in the insect.

## Methods

### Insect strains

The *Tribolium castaneum* GA-1 strain was used for all experiments. Insects were reared in whole wheat flour containing 5% brewer’s yeast at 30 °C under standard conditions as described previously^18^,^19^.

### RNA extraction and cDNA synthesis

Using RNAiso^TM^Plus (TaKaRa), total RNA was isolated from the eggs, larvae, pupae and adults of *T. castaneum*. And 1 μg of total RNA was converted to cDNA by Moloney Murine Leukemia Virus reverse transcriptase (TaKaRa) and an Oligo(dT)_18_ primer (TaKaRa).

### Phylogenetic analysis

With the sequences of *D. melanogaster dy*, *dyl* and *miniature* (http://flybase.org/), we searched the genome databases of these insects, including *T. castaneum* (http://beetlebase.org/), *A. gambiae* (https://www.vectorbase.org/index.php), *B. mori* (http://silkworm.genomics.org.cn/), *A. mellifera* (http://hymenopteragenome.org/beebase/), *A. pisum* (http://www.aphidbase.com/aphidbase/), *P. h. humanus* (https://www.vectorbase.org/index.php) and National Center for Biotechnology Information (NCBI, http://www.ncbi.nlm.nih.gov/guide/). These sequences were aligned with Clustal Omega (http://www.ebi.ac.uk/Tools/msa/clustalo/). Then the Neighbour-joining tree was reconstructed by MEGA 5 using the bootstrap method with 1000 replications (Fig. 1). To test the topology of Neighbor-joining tree, we reconstructed the Maximum likelihood tree by MEGA 5 (Fig. S1).

### qRT-PCR

To check the gene expression profiles, qRT-PCR was performed with FastStart Universal SYBR Green Master (ROX) (Roche). The data are expressed here as the relative mRNA levels normalized to a control gene, *T. castaneum ribosomal protein S3* (*rps3*)^20^, using the ΔΔCT method^21^. Three batches of samples were used for qRT-PCR. The primers were listed in Table 1.

### RNAi

RNAi was performed as previously^9^,^20^. Negative controls consisted of injections of either *vermillion* (*ver*) dsRNA or an equal volume of buffer only (IB). Knock-down levels of the target genes were examined by qRT-PCR at the fifth day after injection. These experiments were performed for three biological replications.

### Statistical analysis

The mean values of the RNAi-treated insects versus the mean values of the control insects were compared using the one-way ANOVA program of SPSS version 13.0. All the data are presented as the mean ± standard error. “*” indicates *p* < 0.05, and “**” indicates *p* < 0.001.

## Additional Information

**How to cite this article**: Li, C. *et al*. Dusky-like is required for epidermal pigmentation and metamorphosis in *Tribolium castaneum*. *Sci. Rep*. **6**, 20102; doi: 10.1038/srep20102 (2016).

## Supplementary Material

Supplementary Information

## Figures and Tables

**Table 1:** List of primers used.

Gene	Sense primer (5′-3′)	Anti-sense primer (5′-3′)	Utility
*dyl*	TAATACGACTCACTATAGGGGTACGGCCAAAGATCATGAG	TAATACGACTCACTATAGGGGCCGACTGAATGACCACA	dsRNA synthesis
	TCACCTTTGGGATATTAACCG	CGCATGTGAGTCTTTTCGCAC	PCR
*ftz-f1*	TAGACAAAACGCAAAGGAAAAGG	GTTTGTACATCGGACCGAATTT	PCR
*svb*	TAATACGACTCACTATAGGGGAAAATACCCCCAAACCACA	TAATACGACTCACTATAGGGTGTCCAGTATCACAATCCATGT	dsRNA synthesis
	GAGGCACACGAGGACGCATA	CTTCAGACAGTGCGATTCCAAC	PCR
*miniature*	TACGAGAAAACTGCCTCCAAAC	CCTCCTCCATCAGACGCGA	PCR
*singed*	TGCTCAAAACGCGGTCTAATAC	TCGTTTCGTGCTCCAATCG	PCR
*forked*	CAGATGTTGAGAGAGGCGGA	GCACTCGTGTGTTTCTTGGATTT	PCR
*blimp-1*	TAATACGACTCACTATAGGGCACGACAACTACCCTAACCA	TAATACGACTCACTATAGGGGCTTCTTACAAATGCCACAC	dsRNA synthesis
	TCTATGCCAAAGATGCGGTAC	TCATTTTGTATTGACAGGCGATTAA	PCR
*rps3*	TCAAATTGATCGGAGGTTTG	GTCCCACGGCAACATAATCT	PCR
