# Supplementary material for: Dusky-like is required for epidermal pigmentation and metamorphosis in Tribolium castaneum
Source: Sci Rep. 2016 Feb 1;6:20102. doi: 10.1038/srep20102 (PMC4735578; doi:10.1038/srep20102)
Supplement: Supplementary Information [file srep20102-s1.pdf]

Dusky-like is required for epidermal pigmentation and metamorphosis in *Tribolium castaneum*

Chengjun Li, Xiaopei Yun, Bin Li*

Jiangsu Key Laboratory for Biodiversity and Biotechnology, College of Life Sciences, Nanjing Normal University, Nanjing 210023, China

Author's email addresses:

lcj3314@163.com; yunxiaopei619@126.com; libin@njnu.edu.cn

*To whom correspondence should be addressed: libin@njnu.edu.cn

Tel/fax: +86-25-85891763

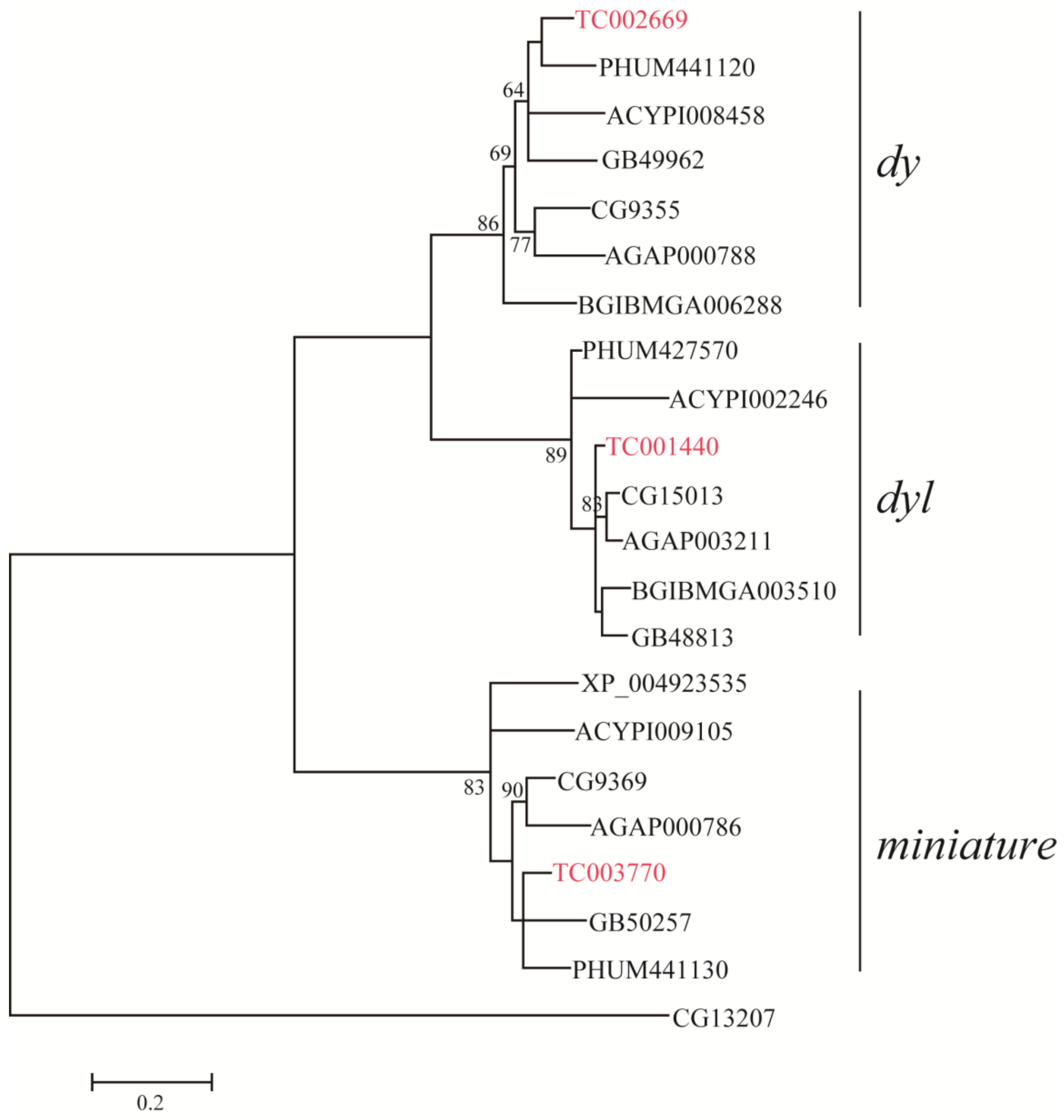

Fig. S1. Phylogenetic tree of insect *dy* and *dyl* by Maximum Likelihood method. TC NO. indicates the *T. castaneum* gene; GB NO. indicates the *A. mellifera* gene; PHUM NO. indicates the *P. h. humanus* gene; ACYPI NO. indicates the *A. pisum* gene; BGIBMGA NO. and XP_004923535 indicate *B. mori* genes; AGAP NO. indicates *A. gambiae* gene; CG NO. indicates the *D. melanogaster* gene. The tree is rooted by *D. melanogaster* no mechanoreceptor potential A (nompA) (CG13207). The bootstrap value below 60% was removed from phylogenetic tree.

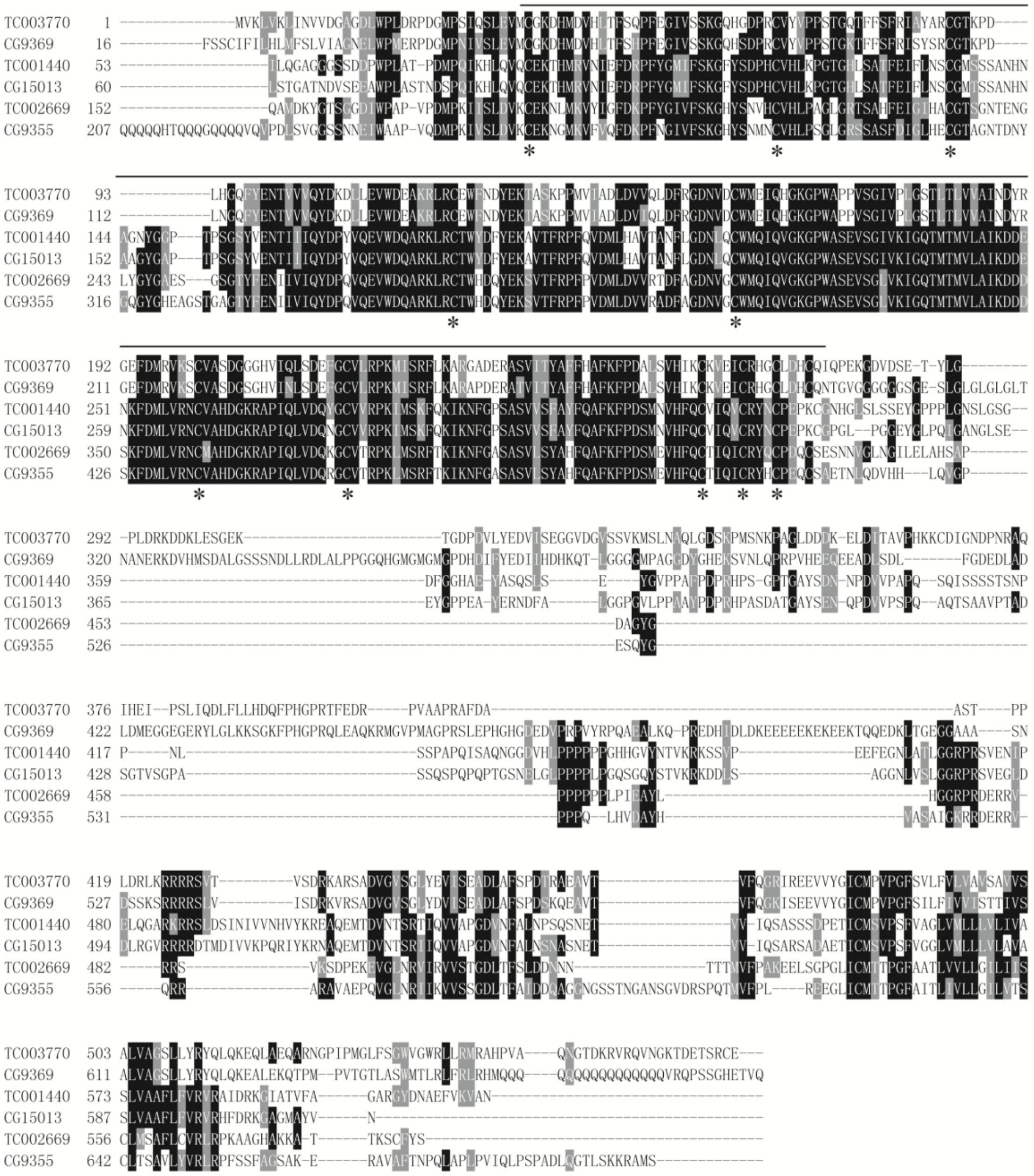

Fig. S2. Sequence alignment of insect Dy, Dyl and Miniature. TC NO. indicates the *T. castaneum* Dy, Dyl and Miniature; CG NO. indicates the *D. melanogaster* Dy, Dyl and Miniature. Black background indicates conserved residues; gray background indicates similar residues. The conserved cysteines are indicated by an asterisk (*) below the alignment

sequence. Zona pellucid (ZP) domain is predicted by smart (http://smart.embl-heidelberg.de/) and marked by single black line on the top of alignment sequence.

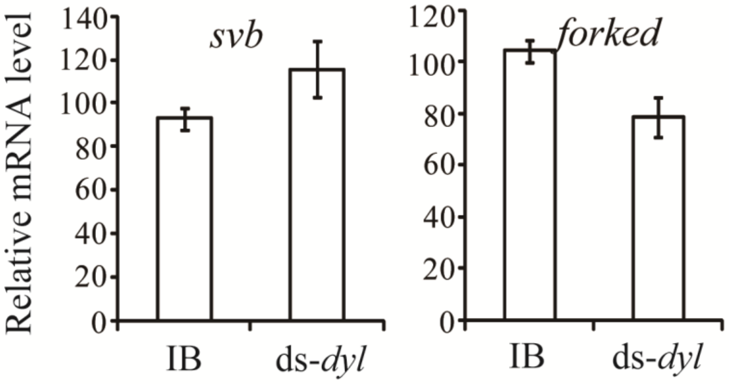

Fig. S3. *Dyl* silencing showed no effects on *svb* and *forked* expressions in *T. castaneum*. Control, beetles that have received no injection; IB, beetles injected with physiological buffer and *ver* dsRNA; ds-*dyl*, beetles injected with *dyl* dsRNA. Three larvae of each group were used to extract total RNA for qRT-PCR analysis.
